# Lacerated Wound of Posterior Perinæum

**Published:** 1875-02

**Authors:** E. T. Henry

**Affiliations:** Vicksburg, Miss.


### LACERATED WOUND OF POSTERIOR PERINÆUM.

BY E. T. HENRY, M.D., VICKSBURG, MISS.

About the 5th of October, 1874, Dr. J. R. Hicks invited me to see Harden Nichols, carpenter, who had been admitted into the city hospital on the 25th of the previous month, with a lacerated wound of the posterior perinæum.

He was driving nails with a claw-hammer in the ceiling of a room; the low scaffolding giving way, he fell violently to the floor, sitting down upon the claw of the hammer, still holding the handle in his hand. The handle must have been between his legs, or nearly so, to have inflicted the wound it did.

In passing through the pants, the claws made two holes, a few of the threads between them not being broken. The wound in the flesh was one, and large enough for two fingers to enter at once. Passing in behind the anus, the course of the claws was upwards and forwards, entering the rectum about an inch above the sphincter, and passing out so as to enter the bladder about the union of the base and posterior portion. Feces and urine passed freely through the wound for some time.

Dr. Hicks, after having the bowel—and bladder, I suppose—well washed with tepid water from a Davidson's syringe, kept the patient on his side, leaning forward as much as possible, with a gum catheter in his bladder, for about a week.

At the time I saw him the external wound had granulated very little, the bowel emptying itself through that opening. Upon introducing the finger into the wound, I could feel it enter the rectum, pass along it some distance and then enter the bladder—the wound in the latter organ being much smaller than at first, the end of the index finger being hardly admitted. Introducing the index finger of the left hand into the rectum through the anus, it met its fellow of the right about an inch within.

The patient had that morning made water naturally. The parts had been kept as clean as the nature of the case would permit, and a little morphine given at night to insure sleep. This was all the medicine that had been applied to, or taken by, the patient. His condition was so good that I advised Dr. Hicks to continue the same plan of treatment, which he did; and I am glad to say with complete success.

Nichols was discharged on the 5th of December perfectly well, and is now pursuing his usual vocation. So much for *vis medicatrix naturæ.* Dr. Hicks claims no credit, except for giving Nature a fair chance to cure the case, which it did.

